# Factors Associated with Vitamin D Deficiency and Their Relative Importance among Indian Adolescents: An Application of Dominance Analysis

**DOI:** 10.1155/2023/4209369

**Published:** 2023-10-17

**Authors:** Akif Mustafa, Chander Shekhar

**Affiliations:** ^1^International Institute for Population Sciences (IIPS), Mumbai, India; ^2^Department of Fertility, International Institute for Population Sciences (IIPS), Mumbai, India

## Abstract

Vitamin D deficiency is a serious issue in developing nations, including India. This study investigates the determinants of vitamin D deficiency among Indian adolescents and assesses their relative importance using dominance analysis. Data from the Comprehensive National Nutrition Survey (CNNS) conducted between 2016 and 2018 were utilized in this study. Vitamin D levels were assessed based on serum 25-hydroxyvitamin D concentration, with a sample size encompassing 13,065 adolescents aged between 10 and 19 years. Backward stepwise multivariate logistic regression was used to identify the correlates of vitamin D deficiency, and the relative importance of these factors was assessed using dominance analysis. The study identified nine predictors that were significantly associated with vitamin D deficiency at a 1% level of significance (*α* = 0.001). Among these factors, sex was found to be the most significant predictor, with female adolescents being 2.66 (95% CI: 95% CI: 2.39–2.96) times more likely to be vitamin D deficient compared to male adolescents. Lifestyle and behavioral factors, such as “sex,” “wealth index,” and “place of residence,” were more dominant in predicting vitamin D deficiency than biological indicators like “BMI” and “serum creatinine.” This underscores the vital role of sunlight exposure in maintaining sufficient vitamin D levels. In summary, this study sheds light on the multifaceted factors contributing to vitamin D deficiency among Indian adolescents, emphasizing the significance of targeted interventions and public health awareness campaigns to mitigate this pressing issue.

## 1. Introduction

Vitamin D deficiency has emerged as a significant global public health concern, impacting more than one billion individuals worldwide. Insufficient serum 25-hydroxyvitamin D levels have been associated not only with musculoskeletal health issues like osteoporosis, rheumatoid arthritis, fractures, and bone metabolism but also with an elevated risk of diverse nonskeletal disorders, including type-1 diabetes mellitus, cardiovascular diseases, infectious diseases, multiple sclerosis, autoimmune diseases, depression, schizophrenia, obesity, and chronic obstructive pulmonary diseases (COPD) [1–4]. However, the exact nature of the relationship between vitamin D and some of these diseases remains incompletely understood. It remains uncertain whether vitamin D deficiency contributes to the development of these disorders or if the disorders themselves lead to vitamin D deficiency [5]. Nonetheless, the accumulating body of evidence linking vitamin D deficiency to a range of health conditions underscores the significance of maintaining sufficient vitamin D levels for overall health and well-being [5].

Vitamin D serves a multitude of critical functions within the body. Its classical role involves the regulation of calcium absorption and the maintenance of mineral homeostasis. Beyond this, it has been found that vitamin D is essential for the absorption of other essential minerals such as iron, zinc, magnesium, and phosphorus [6]. In its active form, vitamin D aids in controlling the release of parathyroid hormone (PTH). Excessive PTH levels can result in bone loss and fragility, elevating the risk of osteoporosis [7]. In addition, emerging evidence suggests that vitamin D may play a role in regulating insulin secretion and glucose levels in the body [8–10]. Furthermore, vitamin D contributes to the generation of antimicrobial peptides, bolstering cellular immunity and aiding in the defense against infections [11]. Recent research has uncovered that vitamin D plays a role in both innate and adaptive immunity, showcasing its potential as an immunomodulator for autoimmune conditions and cancer. Multiple studies have established a connection between a deficiency in vitamin D and the occurrence of psoriasis, autoimmune thyroid disorders, thyroid cancer, and other autoimmune diseases [12–15]. Moreover, vitamin D influences the proliferation and differentiation of various cell types and modulates cell growth [16]. The presence of vitamin D receptors in numerous tissues throughout the body indicates a broader spectrum of clinical and physiological functions for this vital micronutrient [17]. Given the diverse array of functions it fulfills in the human body, it is evident that vitamin D is an essential nutrient for promoting overall health and well-being.

Vitamin D deficiency is an escalating global concern, often receiving insufficient attention. While certain gaps in our understanding of its prevalence persist, research consistently indicates a rising incidence of vitamin D deficiency worldwide. For instance, when considering the threshold of 25(OH)D < 20 ng/ml, it is estimated that 24% of Americans, 37% of Canadians, and 40% of Europeans have insufficient levels of vitamin D [18]. However, in regions such as Africa, the Middle East, and Asia, vitamin D deficiency appears to be even more prevalent [19]. In India, despite the abundance of sunlight in this tropical country, multiple small scale studies have reported a high prevalence of vitamin D deficiency. Estimates of vitamin D deficiency in various population groups in India range from 34% to a staggering 94% [20]. Nevertheless, it is worth noting that these studies have been conducted on relatively small sample sizes and may not be representative of the entire nation.

The adolescent years mark a period of significant physical and mental development, underscoring the importance of maintaining adequate vitamin D levels for overall health and well-being. While previous studies have underscored the prevalent vitamin D deficiency among Indian adolescents, none of these studies have achieved a large-scale, nationally representative scope. In addition, none have undertaken a comprehensive analysis of the anthropometric and biological factors associated with vitamin D deficiency within this population [21]. To bridge these gaps in knowledge, the present study seeks to investigate the demographic, socioeconomic, and anthropometric factors associated with vitamin D levels among Indian adolescents. Furthermore, the study employs a novel dominance analysis approach to evaluate the relative significance of these factors in predicting vitamin D deficiency. To the best of our knowledge, this study represents the world's first use of this approach for analyzing the determinants of vitamin D deficiency.

The aims of this study are twofold. First, it seeks to explore the relationship between vitamin D deficiency and a range of demographic, socioeconomic, anthropometric, and biological factors among adolescents in India. Second, it employs dominance analysis to assess the relative importance of the most significant predictor variables. By achieving these objectives, this study aims to contribute to the understanding of the factors that influence vitamin D status among Indian adolescents, which can inform the development of effective strategies for preventing and addressing vitamin D deficiency in this population.

## 2. Methodology

This study draws upon data obtained from the Comprehensive National Nutrition Survey (CNNS), conducted between 2016 and 2018. This comprehensive survey was undertaken by the Indian Ministry of Health and Family Welfare, with technical support from UNICEF, and data management services were furnished by the Population Council. The CNNS survey encompasses a wealth of information on the nutritional status, body measurements, dietary habits, and micronutrient levels of individuals aged 0–9 years across 30 states in India. The survey adopted a multistage sampling approach to gather data, with a specific focus on three age categories: preschoolers, school-age children, and adolescents. This study, in particular, concentrates on adolescents aged 10–19 years. The original planned national sample size for the survey encompassed 122,100 children and adolescents from 2,035 primary sampling units. This comprised a sample size of 40,700 individuals for household interviews and anthropometric measurements, as well as 20,350 individuals for biological sample collection. It is worth noting that the individual interview response rate was notably high at 95%. However, the response rate for the collection of biological samples was relatively lower, ranging between 63% and 64%. For further details regarding the survey's methodology and data management, readers are encouraged to refer to the CNNS (2016–18) report [22].

### 2.1. Collected Parameters

The information collected in the CNNS can be broadly categorized into three main groups: household interviews, anthropometric measurements, and biological sample collection. In household interviews, data on general socioeconomic and demographic characteristics of the household and information regarding feeding practices and dietary diversity within the household were collected. Anthropometric measurements encompassed the assessment of various characteristics, including height/length, weight, mid-upper arm circumference (MUAC), triceps skinfold thickness (TSFT), and subscapular skinfold thickness (SSFT). The survey also encompassed the collection of data related to a wide range of biological indicators, such as hemoglobin levels, C-reactive protein, serum protein and albumin levels, serum ferritin, serum zinc, serum B12, serum 25(OH)D, urinary iodine levels, blood pressure, HbA1C, serum cholesterol, LDL (low-density lipoprotein), HDL (high-density lipoprotein), triglycerides, and serum creatinine. A list of all the anthropometric and biological predictor variables included in the analysis is shown in Table 1. Detailed information regarding the equipment used and the method of assessment of the abovementioned parameters can be found in the CNNS report.

### 2.2. Sample Size

The planned sample size in the survey for collecting biological samples from adolescents aged 10–19 years was 20,350. However, data on serum vitamin D (the primary outcome variable of this study) were available for only 13,065 adolescents. The reduction in the sample size could be attributed to factors such as nonresponse, subpar data quality, and invalid observations. Consequently, this study relies on a sample size of 13,065 adolescents. To ensure that the findings and estimates accurately represent the entire national population, we applied sample weights from the CNNS dataset.

### 2.3. Blood Collection and Estimation of Serum 25(OH)D

During the data collection process, the study followed established protocols for the collection and estimation of serum 25(OH)D levels. Participants were advised to fast for 8–10 hours prior to blood sample collection, and trained phlebotomists collected 10 ml of venous blood to assess micronutrient status. The samples were transported to the nearest collection center in cooling bags and stored at −20°C until analysis. An antibody competitive immunoassay method with a chemiluminescence (Siemens Centaur) method was used to determine the concentration of serum 25(OH)D in the samples. Standard and extensive measures of internal and external quality control were implemented throughout the entire procedure to ensure the validity and reliability of the data. Both parents and children participated in the study. More detailed information about the collection process can be found in the CNNS (2016–18) report [22].

### 2.4. Ethics Approval and Consent to Participate

The CNNS survey received ethical approval from both the International Review Board of the Population Council and the Ethics Committee of the Postgraduate Institute of Medical and Research. To ensure ethical standards, informed written consent was secured from parents or caregivers of children under 10 years old. For adolescents aged 11–17 years, written informed consent was obtained from both parents or caregivers as well as from the adolescents themselves. Respondents aged above 17 years provided their own written informed consent. These consent procedures were reported by CNN and are elaborated upon in the CNNS report [22].

### 2.5. Outcome Variable

This study focuses on the level of serum 25-hydroxyvitamin D (25(OH)D) as the primary outcome variable, which is considered the most reliable indicator of vitamin D levels in the body [1]. The 25(OH)D concentration is an indicator not only of the amount of vitamin D produced in the skin but also of the amount absorbed through diet. In this study, 25(OH)D concentration was measured using an antibody competitive immunoassay based on the chemiluminescence (Siemens Centaur) method. To define vitamin D deficiency, this study adhered to the criteria set by the Institute of Medicine (IOM), USA, which designates a serum 25(OH)D level of less than 12 ng/ml as indicative of deficiency [22, 23]. For the sake of facilitating statistical analysis, the study established a binary variable, where “deficient” corresponds to 25(OH)D levels less than 12 ng/ml (coded as 1) and “adequate” signifies 25(OH)D levels of 12 ng/ml or higher (coded as 0).

### 2.6. Predictor Variables

#### 2.6.1. Demographic and Socioeconomic Variables

Gender (male, female); place of residence (urban, rural); wealth index (poorest, poorer, middle, rich, and richest); mother's education level; religion (Hindu, Muslim, Sikh, Christian, and others); caste (SC, ST, OBC, and others); age (in months); and region (northern, eastern, western, southern, central, and north-eastern). The “Region” variable not only served as a control for state-level fixed effects but also functioned as a proxy indicator for altitude, thereby accounting for the potential influence of altitude in the analysis.

#### 2.6.2. Anthropometric and Biological Variables

Almost all the anthropometric and biological variables provided in the CNNS dataset were included in the regression analyses as independent variables.

### 2.7. Statistical Analysis

Statistical analyses in this study were performed using STATA-16 software [24]. Backward stepwise multivariate logistic regression was utilized to examine the associations between various factors and vitamin D deficiency. The alpha level for the backward stepwise estimation was set at 1% (0.01), indicating that only predictor variables with strong statistical evidence of an association with vitamin D deficiency were considered. In the regression model, odds ratios were reported solely for predictor variables with a *p* value less than 0.01. It is important to note that the data used in this study had a hierarchical structure, with observations clustered within primary sampling units (PSUs). To account for any potential intracluster correlation, clustered robust standard errors were applied in the regression analysis.

We employed dominance analysis to assess the relative importance of the significant predictors of vitamin D deficiency (correlations identified through regression analysis). This technique is an extension of multiple regression and measures the importance of predictor variables for an outcome variable [25]. The analysis involves creating all possible combinations of predictor variables and running regression against the outcome variable to calculate the *R*^2^ values for pairwise comparison. The *“domin”* and *“moremata”* packages in STATA-16 were used to conduct dominance analysis. Suppose there are “*x*” predictor variables in the analysis, then the technique will make 2*x* − 1 combinations of predictor variables. For instance, if there are five predictor variables, the technique will make 31 combinations of variables and run regression with each combination against the outcome variable to calculate the *R*^2^ values for pairwise comparison.

The *“domin”* package in STATA calculates three statistics: general dominance statistics, conditional dominance statistics, and complete dominance. The general dominance statistics represent the predictor's additional/marginal contribution in the overall fit statistics across all models in which it was included. If a variable *X* has a general dominance statistics greater than variable *Y*, it implies that variable *X* dominates variable *Y*. Conditional dominance statistics are the average incremental contribution to the nth order overall model fit statistics. The nth order refers to the estimation model which has exactly “*n*” predictor variables. If a predictor variable *X* has greater conditional dominance statistics than variable *Y* across all *n* orders, then it indicates that variable *X* conditionally dominates variable *Y*. The general dominance statistics of a predictor variable are the arithmetic mean of all conditional dominance statistics for that variable. A predictor variable *X* completely dominates variable *Y* when the additional contribution of *X* in each subset model fit is greater than the contribution of *Y*. In other words, *X* is more associated with the outcome variable than *Y* in both pairwise comparison and comparison in the presence of all possible combinations of predictor variables. Complete dominance provides the strongest evidence, as it is purely unaveraged and puts each predictor variable against one another in every possible comparison. More detailed information about the technique can be found in STATA's *“domin”* module [25, 26].

## 3. Results

Mean levels of anthropometric and biological parameters are shown in Table 1. The adolescents had a mean BMI of 18.2 (SD = 4.0), a mean hemoglobin level of 12.9 ng/ml (SD = 1.7), an average serum cholesterol level of 140.6 mg/dl (SD = 32.9), and a mean serum 25(OH)D level of 17.1 (SD = 8.2).

Table 2 presents the sociodemographic characteristics of the study participants and the prevalence of vitamin D deficiency across various background variables. Among the participants, 48.6% were female, 56.87% resided in rural areas, and 72.64% identified as Hindu. In terms of socioeconomic status, 35.05% belonged to the Other Backward Classes, 32.09% came from the fifth quantile (richest) of the wealth index, and 23.77% were from the North-Eastern region of India. The prevalence of vitamin D deficiency exhibited significant variations across different subgroups of the study population. Among females, the prevalence of vitamin D deficiency was higher at 35.47%, while urban residents had a prevalence rate of 35.35%. Sikh adolescents showed the highest prevalence rate at 68.91%, whereas adolescents from affluent households had a prevalence rate of 35.91%. Adolescents from the northern region of the country had the highest prevalence rate at 43.93%. Overall, the prevalence of vitamin D deficiency in the Indian adolescent population stood at 24.97%, considering the serum 25(OH)D level cut-off of <12 ng/ml.

Table 3 displays the results of stepwise logistic regression that only included predictor variables with a *p* value less than 0.001, indicating significance at a 99% confidence level. The analysis revealed that the odds of being vitamin D deficient decreased by 5% with each year increase in age (OR: 0.95; 95% CI: 0.93–0.97). Gender disparities were evident, as female adolescents had 2.66 times higher odds of vitamin D deficiency compared to their male counterparts (OR: 2.66; 95% CI: 2.39–2.96). Adolescents residing in urban areas were 1.63 times more likely to be vitamin D deficient than their rural counterparts (OR: 1.63; 95% CI: 1.43–1.87). The analysis also found a positive association between wealth index and vitamin D deficiency (OR: 1.17; 95% CI: 1.11–1.23). This implies that adolescents from affluent households faced a higher risk of vitamin D deficiency compared to those from less affluent backgrounds. Body mass index (BMI) was also found to be significant, with a 5% increase in the odds of vitamin D deficiency for every unit increase in BMI (OR: 1.05; 95% CI: 1.024–1.074). Conversely, there were negative associations observed between vitamin D deficiency and mid-upper arm circumference (MUAC) (OR: 0.96; 95% CI: 0.93–0.98), serum creatinine (OR: 0.28; 95% CI: 0.20–0.98), and glycosylated hemoglobin levels (OR: 0.83; 95% CI: 0.73–0.94). The odds of vitamin D deficiency were found to increase by 4% with each unit increase in TSFT (OR: 1.04; 95% CI: 1.02–1.06). Triceps skinfold thickness (TSFT) displayed a distinct pattern, with a 4% increase in the odds of vitamin D deficiency for each unit increase in TSFT (OR: 1.04; 95% CI: 1.02–1.06). Notably, all other predictor variables, including UV index, did not demonstrate significant associations with vitamin D deficiency at the 99% confidence level (1% level of significance).

As there were 9 predictor variables, a total of 511 (2^9^ − 1) regressions were run by the software to conduct dominance analysis. Summary of general dominance statistics is presented in Table 4. Column 1 of the table shows general dominance statistics; column 2 shows standardized dominance statistics which is general dominance statistic vector normed or standardized to be out of 100%. From the table, it becomes evident that the “sex” of individuals emerged as the most influential predictor of vitamin D deficiency. Remarkably, “sex” alone accounted for the explanation of 41% of the explained variance in the dependent variable. Following closely, serum creatinine assumed the second-most dominant position, contributing to 16.35% of the explained variance in vitamin D deficiency. The wealth index claimed the third most vital position among the variables, while triceps skinfold thickness (TSFT) and place of residence occupied the 4th and 5th ranks in terms of dominance. Glycosylated hemoglobin was found to be the least dominant factor in explaining vitamin D deficiency.

Table 5 shows the conditional dominance among the predictor variables. The first column of the table displays the average marginal contribution to the overall model fit statistic with 1 independent variable in the model; similarly, the second column shows the average marginal contribution to the overall model fit statistic with 2 independent variables in the model, and so on. Here also, sex was conditionally dominant on all the predictor variables. TSFT was dominant over serum creatinine at 1^st^ and 2^nd^ orders, but at higher orders, serum creatinine was significantly dominant on TSFT. Glycosylated hemoglobin was found to have the least importance in predicting vitamin D deficiency.

Complete dominance designations are presented in Table 6. The rows of the table correspond to the dominance of the independent variable in that row over the independent variable in each column. The value “1” indicates that the independent variable associated with the row completely dominates the independent variable associated with the column. On the other hand, the value “−1” indicates that the independent variable associated with the row is completely dominated by the independent variable associated with the column. The value “0” indicates that there is no complete dominance relationship between the independent variable associated with the row and the independent variable associated with the column. From the first row, we can conclude that “Age” is completely dominated by “sex,” “wealth index,” and “serum creatinine.” The second row shows that “sex” is completely dominant over all the eight predictor variables. The third row of the table depicts that “area” completely dominates “BMI” and “glycosylated hemoglobin” and is completely dominated by “sex” and “wealth index.”

## 4. Discussion

This study aims to evaluate the key predictors that significantly influence vitamin D deficiency among adolescents in India. The results indicate that several variables, including “sex,” “serum creatinine,” “wealth index,” and “place of residence,” emerge as the most influential factors linked to vitamin D deficiency among adolescents. In addition, the study underscores the significance of BMI, TSFT, and age as other important determinants of vitamin D deficiency in this demographic.

Among the selected predictors, “sex” emerged as the most dominant factor in predicting vitamin D deficiency. The study findings revealed significantly higher odds of vitamin D deficiency among female adolescents in comparison to their male counterparts, a trend consistent with prior research [27–31]. The body's production of vitamin D3 relies on exposure to ultraviolet B (UVB) radiation from sunlight [1, 32]. However, various individual factors, such as skin pigmentation, clothing habits, and the use of sunscreen creams, as well as sun-avoidant lifestyles, can influence the extent of cutaneous UVB exposure. The divergence in vitamin D levels between males and females can be attributed to differences in lifestyle and behavior. Males typically engage more in outdoor activities, which may account for their higher serum 25(OH)D concentration. In addition, the use of sunscreen products and sun-avoidance behaviors tend to be more prevalent among females [27]. It is important to note that gender-based discrimination and a cultural preference for sons are widespread in India, factors that could also contribute to the lower levels of vitamin D observed among female adolescents compared to males [33].

Creatinine is a byproduct generated when muscles metabolize creatine, a compound found in the body. It circulates in the bloodstream and is filtered by the kidneys for elimination through urine. Serum creatinine levels serve as indicators of both kidney function and muscle mass. Elevated levels of serum creatinine are indicative of kidney disease, as the kidneys' ability to filter it decreases with declining kidney function [34]. Interestingly, this study identified a negative association between vitamin D deficiency and serum creatinine levels, implying that adolescents with higher serum creatinine levels exhibited lower rates of vitamin D deficiency. Dominance analysis further underscored the significance of serum creatinine as the second most important predictor of vitamin D deficiency. A study conducted in the United States also reported a similar association, linking vitamin D activation to increased serum creatinine levels and decreased estimated glomerular filtration rates [35]. However, further research is necessary to gain a deeper understanding of this complex relationship.

As per the study results, the “Wealth Index” emerged as the third most dominant predictor of vitamin D deficiency. In line with prior research findings, the wealth index displayed a positive association with vitamin D deficiency, indicating that individuals with greater wealth were more likely to experience vitamin D deficiency compared to those with lower socioeconomic status [36–38]. This observation can be attributed to lifestyle disparities between affluent and less affluent individuals. Individuals with higher socioeconomic status often lead more sedentary lives and may engage less in outdoor activities that expose them to sunlight. Conversely, adolescents from lower socioeconomic backgrounds tend to participate in outdoor activities and have prolonged exposure to sunlight, possibly due to their involvement in work alongside their parents [36–39]. These lifestyle distinctions can contribute to the varying prevalence of vitamin D deficiency among wealth-related categories.

Triceps skinfold thickness (TSFT) and body mass index (BMI) are commonly used indicators to assess the nutritional status and fat reserves of the body in public health research. The study finds a positive association between both BMI and TSFT and vitamin D deficiency among adolescents in India, meaning that those with higher BMI and TSFT levels have lower serum 25(OH)D concentration compared to those with lower BMI and TSFT. This aligns with previous research that has also established a negative association between BMI and 25(OH)D [37, 40–42]. This can be explained by the fact that high body fat content, which is associated with high BMI and TSFT levels, acts as a reservoir of vitamin *D*, leading to its sequestration in adipose tissues, thus reducing the amount of vitamin D available in the bloodstream. The release of vitamin D from fat is slow, resulting in a decreased concentration of serum 25(OH)D in individuals with a high percentage of body fat [43, 44]. In addition, volumetric dilution may also account for the variability in serum 25(OH)D concentration due to overweight and obesity [45].

As outlined in the study, adolescents residing in urban areas exhibit a higher likelihood of vitamin D deficiency in comparison to their rural counterparts. This trend is likely attributable to several factors. Rural residents often benefit from increased sunlight exposure due to agricultural work and engagement in outdoor activities. Conversely, urban residents tend to limit their sunlight exposure, and they also face elevated levels of environmental pollution. This environmental pollution reduces the amount of UV radiation that reaches their skin, further exacerbating the risk of vitamin D deficiency. Furthermore, the study did find associations between both mid-upper arm circumference (MUAC) and glycosylated hemoglobin and vitamin D deficiency. However, these associations were not as robust as other factors. In fact, these predictors were either completely or conditionally dominated by other covariates, indicating their minimal contribution to explaining the variability in vitamin D deficiency. To highlight their limited impact, the conditional dominance statistic at the 9th order was zero for glycosylated hemoglobin and 0.0001 for MUAC. This underscores their relatively small role in influencing vitamin D deficiency.

Age was identified as the 6th most significant factor associated with vitamin D deficiency in Indian adolescents. However, upon examining the complete dominance table, it was found that the impact of age on vitamin D deficiency is entirely overshadowed by “sex,” “serum creatinine,” and “wealth index.” This means that regardless of age, females and adolescents with higher socioeconomic status are more likely to be at risk for vitamin D deficiency.

This study provides valuable insights into the prevalence of vitamin D deficiency among Indian adolescents. It underscores the role of lifestyle and individual-level factors in contributing to this issue, offering a comprehensive understanding of high-risk groups susceptible to vitamin D deficiency within this population. Based on these findings, several policy recommendations can be proposed as follows:

- Public awareness programs: Implement awareness programs aimed at educating the public about the significance of vitamin D and appropriate sunlight exposure for maintaining good health. These programs should include guidance on optimal times for sunlight exposure to ensure adequate vitamin D levels while minimizing the risk of harmful UV ray exposure.
- Targeted supplementation: Consider vitamin D supplementation for high-risk groups, particularly females and individuals from higher socioeconomic backgrounds. Supplementation can take the form of capsules, tablets, or drops to address deficiencies effectively.
- School-level initiatives: Develop structured school-level programs that incorporate outdoor physical activities into the school day. This initiative can help combat vitamin D deficiency among adolescents by promoting increased exposure to sunlight and physical activity. The study's methodology and findings serve as a foundational resource for future research on vitamin D deficiency in India, offering valuable insights for addressing and mitigating this public health concern.

We believe that this study represents a novel approach to understanding vitamin D deficiency and can serve as a cornerstone for future research endeavors on this topic within India.

## 5. Limitations

The current study is not without limitations, and these limitations should be addressed in future data collection for the CNNS. One of the major limitations is the lack of data on important aspects related to vitamin *D*. For instance, the amount of time spent in sunlight is a critical factor that affects vitamin D levels, but the CNNS does not provide this information. The absence of this information makes it challenging to adequately control for sunlight exposure in regression analyses, potentially introducing omitted variable bias. In addition, calcium intake is strongly associated with vitamin D levels, but it could not be included in the analysis due to the unavailability of data. This omission could limit the study's ability to draw conclusive insights regarding the significance of vitamin D predictors. To enhance the reliability and validity of conclusions about vitamin D deficiency predictors, it is essential that future studies, including those conducted within the CNNS, collect comprehensive data encompassing all relevant factors related to vitamin D levels. This approach will provide a more accurate understanding of the determinants of vitamin D deficiency.

## Figures and Tables

**Table 1:** Selected anthropometric and biological variables included in the regression analysis as independent variables.

	Tool/method used for measurement	Indicator of	Mean ± SD
*Anthropometric variables*			
Body mass index (BMI)	Digital SECA scales and three-piece wooden height/length boards	Overweight and obesity	18.16 ± 4.03
Height for age (*z*-score)	Digital SECA scales and three-piece wooden height/length boards	Stunting, wasting	
Mid-upper arm circumference (MUAC) (cm)	MUAC tape	Nutritional status	21.95 ± 3.65
Triceps skinfold thickness(TSFT) (cm)	Holtain skinfold calipers	Nutritional status	9.81 ± 4.66
Subscapular skinfold thickness (SSFT) (cm)	Holtain skinfold calipers	Nutritional status	8.86 ± 4.16
Waist circumference, (cm), cm	Fiber glass tapes	Abdominal obesity	63.85 ± 9.10

*Biological variables*			
Hemoglobin level (g/dl)	Cyanmethemoglobin method	Anemia status	12.93 ± 1.75
Serum ferritin (*μ*g/l)	Direct chemiluminescence method	Iron deficiency	42.13 ± 38.15
Serum retinol (*μ*g/dL)	HPLC reversed-phase chromatography	Vitamin A deficiency	32.64 ± 34.27
Serum zinc (*μ*g/dL)	(Not disclosed)	Zinc deficiency	78.46 ± 15.89
Serum vitamin B12 (ng/ml)	Competitive immunoassay using direct chemiluminescence	Vitamin B12 deficiency	292.68 ± 141.55
Urine iodine (*μ*g/l)	Microplate method based on the Sandell-Kolthoff reaction	Iodine status	238.65 ± 165.71
Fasting plasma glucose (mg/dl)	Spectrophotometry	Diabetes	90.96 ± 12.88
Serum cholesterol (mg/dl)	Plasma lipid and lipoprotein levels	Cholesterol level	140.61 ± 32.89
Glycosylated hemoglobin (%)	Spectrophotometry	Diabetes	5.23 ± 0.4
Serum creatinine (mg/dl)	Spectrophotometry	Renal function	0.64 ± 0.24
Serum erythrocyte folate (ng/ml)	Competitive immunoassay using direct chemiluminescence	RBC folate deficiency	221.89 ± 191.46

**Table 2:** Distribution of sample population with respect to demographic and socioeconomic categories and prevalence of vitamin D deficiency in respective categories.

	*n*	%	Vitamin D deficiency (%)
Age			
10–14	7,022	53.75	26.33
15–19	6,043	46.25	23.34
Sex			
Male	6,715	51.4	14.82
Female	6,350	48.6	35.47
Place of residence			
Rural	7,430	56.87	21.94
Urban	5,635	43.13	35.35
Religion			
Hindu	9,490	72.64	23.23
Muslim	1,466	11.22	31.31
Christian	1,323	10.13	17.39
Sikh	326	2.5	68.91
Others	460	3.52	24.2
Caste			
SC	2,536	20.93	27.6
ST	2,367	19.53	15.13
OBC	4,247	35.05	23.6
General	2,967	24.49	30.28
Wealth index			
Poorest	1,047	8.01	19.82
Poor	1,707	13.07	18.92
Middle	2,613	20	21.12
Rich	3,505	26.83	29.49
Richest	4,193	32.09	35.91
Region			
North	2,870	21.97	43.93
Central	1,329	10.17	19.98
East	2,333	17.86	27.25
West	1,350	10.33	26.23
South	2,078	15.91	14.57
North-east	3,105	23.77	13.95

**Table 3:** Results of backward stepwise logistic regression model assessing adjusted odds ratios of vitamin D deficiency among Indian adolescents.

	Adj. odds ratio	95% CI
Age	0.95	0.93–0.97
Sex		
Male	1	—
Female	2.66	2.39–2.96
Place of residence		
Rural	1	—
Urban	1.63	1.43–1.87
Wealth index	1.17	1.11–1.23
BMI	1.05	1.024–1.074
MUAC	0.96	0.93–0.98
TSFT	1.04	1.02–1.06
Serum creatinine	0.28	0.20–0.98
Glycosylate hemoglobin	0.83	0.73–0.94

*Note*. BMI: body mass index; MUAC: mid-upper arm circumference; TSFT: triceps skinfold thickness.

**Table 4:** General dominance table.

	Dominance statistics	Standardized dominance statistics	Ranking
Age	0.0047	0.0364	6
Sex	0.0528	0.4131	1
Place of residence	0.0091	0.0712	5
Wealth index	0.0173	0.1349	3
Body mass index	0.0037	0.0286	7
MUAC	0.0022	0.0172	8
TSFT	0.017	0.1332	4
Serum creatinine	0.0209	0.1635	2
Glycosylated hemoglobin	0.0002	0.0019	9

**Table 5:** Conditional dominance table.

	Number of independent variables in the model (order)								
	1	2	3	4	5	6	7	8	9
Age	0.0048	0.0067	0.0067	0.0058	0.0049	0.004	0.0034	0.0029	0.0026
Sex	**0.072**	**0.067**	**0.0614**	**0.0561**	**0.0513**	**0.047**	**0.0434**	**0.0401**	**0.0373**
Area	0.0171	0.0143	0.0119	0.0099	0.0082	0.0069	0.0057	0.0046	0.0035
Wealth index	0.0261	0.023	0.0202	0.0179	0.0161	0.0146	0.0134	0.0124	**0.0115**
Body mass index	0.0057	0.0069	0.0061	0.0047	0.0033	0.0022	0.0016	0.0013	0.0012
MUAC	0.0003	0.0037	0.0044	0.0039	0.003	0.0022	0.0014	0.0007	0.0001
TSFT	**0.0347**	0.0317	0.0267	0.0212	0.0158	0.0111	0.0071	0.0038	0.0012
Serum creatinine	**0.0318**	0.0304	0.0272	0.0235	0.02	0.017	0.0146	0.0126	0.011
Glycosylated hemoglobin	0.0004	0.0004	0.0004	0.0003	0.0003	0.0002	0.0001	0.0001	0.000

The bold values are those values which are found substantially dominant in the conditional dominance. For example sex, the conditional dominance statistics are highest for sex at each order.

**Table 6:** Complete dominance table.

	Age	Sex	Area	Wealth index	Body mass index	MUAC	TSFT	Serum creatinine	Glycosylated hemoglobin
Age	0	−1	0	−1	0	0	0	−1	0
Sex	1	0	1	1	1	1	1	1	1
Area	0	−1	0	−1	1	0	0	0	1
Wealth index	1	−1	1	0	1	1	0	0	1
Body mass index	0	−1	−1	−1	0	0	0	−1	1
MUAC	0	−1	0	−1	0	0	−1	−1	0
TSFT	0	−1	0	0	0	1	0	0	1
Serum creatinine	1	−1	0	0	1	1	0	0	1
Glycosylated hemoglobin	0	−1	−1	−1	−1	0	−1	−1	0

## Data Availability

Data used for this study can be accessed from Population Council (India) on request basis.

## Abbreviations

CNNS: Comprehensive National Nutrition Survey
25(OH)D: 25-Hydroxyvitamin D
BMI: Body mass index
MUAC: Mid-upper arm circumference
SSFT: Subscapular skinfold thickness
TSFST: Triceps skinfold thickness
ng/ml: Nanograms per milliliter
nmol/liter: Nanomoles per liter
SC: Scheduled caste
ST: Scheduled tribe
OBC: Other backward category
PSU: Primary sampling unit.
